# Effects of hydroxychloroquine and favipiravir on clinical course in outpatients with COVID-19

**DOI:** 10.3906/sag-2101-146

**Published:** 2021-06-23

**Authors:** Şeyda Kayhan ÖMEROĞLU, Fehminaz TEMEL, Dilek ALTUN, Burak ÖZTOP

**Affiliations:** 1 İzmir Provincial Health Directorate, İzmir, Turkey; 2 Republic of Turkey, Ministry of Health, Ankara, Turkey

**Keywords:** COVID-19, hydroxychloroquine, favipiravir, outpatients, treatment

## Abstract

**Background/aim:**

Due to the importance of early outpatient treatment to prevent hospitalization and disease progression, we examined the effects of hydroxychloroquine and favipiravir, which were initiated in early period, on the clinical course of COVID-19 outpatients.

**Materials and methods:**

Data of confirmed COVID-19 outpatients over a 4-month period were analyzed retrospectively. Public Health Management System (HSYS) was used for the case-based follow-up. Patients on antiviral therapy for at least five days, including hydroxychloroquine and / or favipiravir and patients who were followed-up for 30 days were included in this analysis.

**Results:**

We enrolled 1489 patients in this study. Overall, 775 (52%) patients were male and a mean age of patients was 38.9 ± 11.1 years. Of these patients, 537 of them were received favipiravir, 545 of them were received hydroxychloroquine and 407 of them were received both favipiravir and hydroxychloroquine. Symptoms improvement on the 14th day of follow-up was 1.8 times higher in the group of patients receiving hydroxychloroquine compared to patients who received favipiravir (p = 0.003). On the 3rd day of follow-up, PCR negativity rate was higher in patients who received hydroxychloroquine (p = 0.004). Hospitalization rates were similar in patients receiving favipiravir and hydroxychloroquine (p = 0.144). However, in the presence of pneumonia at the time of diagnosis, the hospitalization rate was 6.6 times higher in patients who received favipiravir than those who received hydroxychloroquine.

**Conclusion:**

The subgroups of patients treated with hydroxychloroquine and/or favipiravir did not have similar disease severities in our study. Therefore, further studies with homogeneous patient groups to be arranged prospectively are needed.

## 1. Introduction

The coronavirus disease 2019 (COVID-19) pandemic is the biggest health threat to humanity after the 1918 influenza pandemic. Since its first detection in December 2019, COVID-19 has affected nearly 75 million people in a year, and more than 1.5 million people have died due to SARS-CoV-2. SARS-CoV-2 virus causes COVID-19 disease and severe acute respiratory syndrome. The disease is characterized by flu-like symptoms. Some patients are asymptomatic, pulmonary involvement often develop in symptomatic patients and progressive disease can be life threatening^1^.

There is no specific drug to treat COVID-19. Since the beginning of the pandemic, many drugs such as favipiravir, remdesivir, hydroxychloroquine, lopinavir / ritonavir have been used in the treatment of the disease. Chloroquine is a classic antimalarial drug and also has anti-inflammatory and immunomodulatory effects in viral infections. Hydroxychloroquine is a chloroquine metabolite and is less toxic. Chloroquine and hydroxychloroquine have been demonstrated to inhibit SARS-CoV-2 in vitro, but their clinical efficacy and benefits are not yet known. Favipiravir is a nucleotide analogue approved in China and Japan for the treatment of influenza. Favipiravir prevents the replication of the RNA virus by selectively inhibiting RNA-dependent RNA polymerase. Since the RNA-dependent RNA polymerase gene of SARS-CoV-2 is similar to influenza viruses, it has also been proposed in the treatment of COVID-19 [1]. Hydroxychloroquine and favipiravir have been used alone or in combination in the treatment of COVID-19 since the beginning of the pandemic in Turkey. In this study, we aimed to investigate the effects of hydroxychloroquine and favipiravir on the clinical course in COVID-19 outpatients receiving early treatment.

## 2. Materials and methods

The study was carried out in the hospitals located in İzmir, Turkey. This observational retrospective study was approved by the Clinical Research Ethics Committee of İzmir Katip Çelebi Üniversitesi, Institutional Review Board. Data were collected from August 1st to November 30th from all outpatients diagnosed with definitive COVID-19 and receiving treatment. Reverse transcriptase polymerase chain reaction (RT-PCR) test was applied to all outpatients meeting the definition of the possible case definition according to The Republic of Turkey Ministry of Health COVID-19 guideline^2^. PCR sampling frequency was a part of the routine follow-up process. Patients with PCR-documented SARS-CoV-2 RNA from nasopharyngeal samples were prescribed hydroxychloroquine (200 mg twice daily for 5 days) and favipiravir (two 1600 mg oral loading doses on day 1, followed by 600 mg twice daily on days 2–5) as early treatment, whether or not they had symptoms. Serum electrolyte analysis and electrocardiogram were evaluated before the treatment.

Public Health Management System (Halk Sağlığı Yönetim Sistemi-HSYS) was used for the case-based follow-up. Patients who received antiviral treatment for at least five days and patients who were followed-up for 30 days were included in this analysis. All PCR-positive COVID-19 outpatients whose data could be accessed during the study period were included in the study. A total of 468 PCR-positive outpatients were excluded from the study due to being under 18 years of age, previous COVID-19 diagnosis, pregnancy and missing data. PCR samplings were repeated on the 1st, 3th, and 14th days. Symptoms were evaluated on the 1st, 3th, 5th, 14th, and 30th days of follow-up. Demographics and laboratory results on the day of admission were recorded. COVID-19 pneumonia was confirmed by thoracic computed tomography (CT). The clinical data (symptoms, antiviral-related side effects, requirement for hospitalization, recovery) of these outpatients who were followed up by telephone or personal visits were evaluated retrospectively. The treatment was decided by the filiation teams according to the age and comorbid status of the outpatients. Patients were divided into three groups according to the treatment they received such as hydroxychloroquine, favipiravir and both hydroxychloroquine + favipiravir. The primary outcomes were symptom improvement, PCR negativity and need for hospitalization. The effects of hydroxychloroquine and favipiravir on the clinical course were evaluated statistically.

Statistical calculations were performed using SPSS (Statistical Package for the Social Science; IBM Corp, Armonk, NY, USA), version 26. Categorical variables between groups were compared with the chi-square test and confidence interval. ANOVA F test or student-t test was used for the analysis of continuous variables, where appropriate. Logistic regression analysis was performed to explore which factors were predictive for clinical course. Significant variables at p < 0.05 in univariate analyses were introduced in the initial multivariate model. A stepwise approach was applied to evaluate the iteration of variables and to control potential confounders. A p value less than 0.05 was considered statistically significant.

## 3. Results

### 3.1. Demographic findings

During the study period, a total of 1957 outpatients test results were positive for SARS-CoV-2 RNA. Of these outpatients, 468 of them were excluded from the study due to being under 18 years of age, previous COVID-19 diagnosis, pregnancy, and missing data. Finally, we enrolled 1489 outpatients in this study. Overall, 775 (52%) patients were male and the mean age of patients was 38.9 ± 11.1 years. The minority of the patients (11.6%) had at least one comorbid disease including diabetes (10.1%), hypertension (3.2%), and malignancy (0.6%). The proportion of asymptomatic patients was 720 (48.4%), while the number of patients with upper respiratory tract infection was 644 (43.3%), and the number of patients with lower respiratory tract infection was 230 (15.4%). Demographic and laboratory findings are presented in Table 1.

Of these patients, 537 of them received favipiravir, 545 of them received hydroxychloroquine, and 407 of them received both favipiravir and hydroxychloroquine. At least one side-effect attributed to the favipiravir and/ or hydroxychloroquine was observed in 17.5% of patients. The side-effects of antiviral treatment were gastrointestinal disturbances (12.1%), allergic reactions (3.5%), and cardiac arrhythmia (1%). No significant difference was observed between patients receiving favipiravir and hydroxychloroquine in terms of side effects (p = 0.086).

### 3.2. Effects of antivirals on symptom improvement

The rates of symptoms were similar in symptomatic patients except for the loss of smell and taste on the first day of treatment (Table 1). Cough was less common in the group of patients who received hydroxychloroquine on the 3rd day of treatment (p = 0.029). In addition, the group that received hydroxychloroquine had a higher rate of recovery from joint pain on the 14th day and myalgia on the 30th day (p = 0.001 and p = 0.046). The number of patients whose all symptoms improved on the 14th day of follow-up was higher in the group of patients receiving hydroxychloroquine (p = 0.003).

Logistic regression model was used to examine the independent effects of antivirals on symptom improvement. The antiviral drugs, the presence of comorbidity, and pneumonia are included in this model. On the 14th day of follow-up, regardless of the presence of comorbidity and pneumonia, symptom improvement was 1.8 times higher among patients who received hydroxychloroquine compared to patients who received favipiravir (Table 2).

### 3.3. Effects of antivirals on PCR negativity

On the 3rd day of follow-up, PCR negativity rate was higher in patients receiving hydroxychloroquine (p = 0.004), but this difference was not significant on the 14th day (p = 0.502). The presence of pneumonia as a confounding factor was included in the logistic regression model to examine the independent effects of antivirals on PCR negativity at 3rd day of follow-up. Sex, age, and comorbidity were not included in this analysis because they were not found to be related with PCR negativity. Accordingly, on the 3rd day of the treatment, it was found that PCR negativity was higher in the asymptomatic patients (Odds ratio [OR]: 1.41, 95% Confidence Interval [CI]: 1.12–1.79), in patients without pneumonia (OR: 1.57, 95% CI: 1.11–2.24) and in patients who received hydroxychloroquine compared to patients received favipiravir (OR: 1.44, 95% CI: 1.09–1.91). Combined antiviral therapy had no effect on PCR negativity on the 3rd day of treatment (Table 3).

### 3.4. Subgroup analysis of hospitalized patients

All patients included in the study recovered from COVID-19 during the follow-up period. A total of 40 patients were hospitalized; 7 of them had severe pneumonia, 5 of them were followed in Intensive Care Unit (ICU), and only one patient required mechanical ventilation. Hospitalization rates were similar in patients receiving favipiravir and hydroxychloroquine (p = 0.144) (Table 1).

Logistic regression analysis was used for evaluating independent factors affecting hospitalization. The presence of pneumonia was included in the model as an effect modifier. The odds ratio for age indicated that each 1-year increase in age increased the relative risk of hospitalization by 1.04-fold (Table 4). In the presence of pneumonia at the time of diagnosis, the hospitalization rate was 6.6 times higher in patients who received favipiravir than those who received hydroxychloroquine. In similar, the hospitalization rate was 7.3 times higher in patients who received combined therapy compared with those who received hydroxychloroquine (Table 4).

## 4. Discussion

Aside from the now, more than 1.8 million Turkish people have been infected with SARS-Cov-2 according to the Republic of Turkey Ministry report**. Although social distancing, staying at home and wearing face masks served to reduce the hospital burdens and spread them over time, these measures only reduced reproduction numbers to about 1.0 [2]. Moreover, considering that isolation policies will be lifted over time, it can be predicted that many people will be exposed to the COVID-19 in the future, even if these social isolation measures are maintained.

While the vast majority of COVID-19 patients is at low risk of progression or show the infection without symptoms, for the remaining patients, outpatient treatment is necessary for preventing hospitalization and disease progression. Thus, the way to prevent loss of life and return the society with normal functioning is an effective and safe outpatient treatment [3].

Although there is no anti-viral treatment with proven efficacy in the treatment of COVID-19, drugs such as chloroquine/hydroxychloroquine, azithromycin, lopinavir/ritonavir, favipiravir and remdesivir have been used widely. Hydroxychloroquine and favipiravir, which are included in the treatment protocol in many national treatment guidelines, are also used in our country according to The Republic of Turkey Ministry of Health COVID-19 guideline**. There are many studies about these drugs in the literature especially on hospitalized patient groups [4–7]. Early outpatient disease is not the same as a later hospitalization illness, as a result, different treatments’ affects may be obtained in different patient groups [3]. Due to the limited number of studies with large numbers of outpatients examining early initiation of antiviral therapy, we examined the effects of these drugs, which were initiated in early period, on the clinical course of outpatients.

Chloroquine and its derivatives are widely used as immunomodulators in the treatment of rheumatic diseases [8]. As the pharmacological property of chloroquine and its derivatives are studied, additional clinical applications, especially based on its antiviral activity against human coronaviruses are increasingly appreciated [9]. The action of hydroxychloroquine such as antioxidant activities and regulation in proinflammatory cytokines encourages its administration due to the cytokine storm in patients with severe COVID-19 [10]. Therefore, hydroxychloroquine has been used in the treatment with the assumption that it will be a protective agent in SARS-CoV-2 infection with its antiviral and autoimmune regulating effect. While hydroxychloroquine has been found to be effective in the prophylaxis and treatment of COVID-19 in some studies, other studies have claimed otherwise [7,11,12]. In a meta-analysis conducted by Elavarasi et al., there was no significant difference in virologic clearance between placebo and hydroxychloroquine in the meta-analysis of two randomized-controlled trials and three cohort studies [11]. In addition, the time of fever remission, clinical deterioration, development of ARDS and need for mechanical ventilation rates were similar between the hydroxychloroquine arm and standard of care [11]. In a meta-analysis in which four randomized controlled trials were analyzed, the use of hydroxychloroquine for COVID-19 prophylaxis, compared to placebo, did not reduce the risks of developing COVID-19, hospitalization or mortality; however, hydroxychloroquine use increased the risk of adverse events [12]. In our study, hydroxychloroquine was found effective in improving symptoms. We also showed COVID-19 outpatients who received hydroxychloroquine had higher rate of PCR negativity on the 3rd day, but no significance was found in terms of PCR negativity on the 14th day. In addition, the use of hydroxychloroquine was associated with fewer hospitalization rates in COVID-19 outpatients with pneumonia. Our results may be due to the higher rate of asymptomatic or mildly symptomatic patients in the group receiving hydroxychloroquine. Eventually, the fact that the patient groups were not randomly selected prospectively and the absence of a control group make the generalizability of our research findings debatable. In addition, subgroups of patients treated with hydroxychloroquine and/or favipiravir did not have similar disease severities in our study. The higher rate of pneumonia at the time of diagnosis in patients receiving favipiravir may explain the higher rate of hospitalization in this patient group. Therefore, further studies with homogeneous patient groups to be arranged prospectively are needed.

Favipiravir, a potent inhibitor of RNA-dependent RNA polymerase, was approved for reemerging pandemic influenza in Japan [13]. Favipiravir has shown in vitro activity against SARS-CoV-2 by reduction in the number of infectious particles and cytopathic effect [14]. There are mostly observational studies evaluating the efficacy of favipiravir in different patient groups, and conflicting results have been obtained in these studies [15,16]. Two randomized, open-label controlled trials are currently underway to evaluate the efficacy of early favipiravir treatment in outpatients with early stage COVID-19 [17,18]. In our study, the parameters used to evaluate clinical course, were yielded more positive outcomes in patients receiving hydroxychloroquine compared to patients receiving favipiravir. The positive effects of early antiviral therapy on the clinical course may be attributed to the anti-inflammatory and immunomodulatory effects rather than the antiviral effect. A possible explanation for the superiority of favipiravir over combination therapy in our study can be attributed to the fact that the number of asymptomatic patients in the patient group receiving favipiravir is higher compared to the patient group receiving combination therapy.

Our study has some limitations. The first of these was the absence of a control group that did not receive antiviral treatment. Secondly, since we did not have complete access to the entire number of patients diagnosed during the study period, it is difficult to assess whether our findings were representative of the entire population. Similarly, we could not access any information about the quality control of the data. Another limitation of the study was that the different subgroups of patients treated with different medications had different disease severities, and that it is difficult to make comparisons of the effectiveness of these drugs. In addition; observational, retrospective and nonrandomized design of our study restricted the reliability of our findings, even though large number of patients and multicentric design.

The antiviral drugs administered in the early phase of infection can shorten the course of the clinical disease and thus may reduce the infectiousness by reducing viral spread. Data of the ongoing randomized controlled trials and meta-analyses will indicate the efficacy of these antivirals in COVID-19 outpatient clearly, so we need to wait for more clinically valid evidence to confirm the value of this antiviral agent for COVID-19 treatment.

## Figures and Tables

**Table 1:** Demographic, laboratory, and follow-up findings of COVID-19 outpatients.

	Total (n = 1489. 100%)	Favipiravir (n = 537. 36.1%)	Favipiravir +hydroxychloroquine (n = 407, 27.3%)	Hydroxychloroquine (n = 545. 36.6%)	P value

**Male (n. %)**	775 (52)	278 (51.8)	210(51.6)	287 (52.7)	0.941

**Age (years)** (Mean ± SS / Median min–max)	38.9 ± 11.1	39.7 ± 11.2	39 ± 10.8	37.9 ± 11.1	**0.021**
	39 (16–82)	40 (18–82)	39 (18–77)	37 (16–80)	

**Comorbidity (n, %)**					

At least one underlying disease	172 (11.6)	58 (10.8)	51(12.5)	63 (11.6)	0.723

Diabetes mellitus	150 (10.1)	51 (9.5)	46 (11.3)	53 (9.7)	0.617

Hypertension	48 (3.2)	12 (2.2)	16 (3.9)	19 (3.5)	0.293

Malignancy	9 (0.6)	1 (0.2)	1 (0.7)	5 (0.9)	0.324

Symptoms at the time of diagnosis (n. %).					

Asymptomatic patients	720 (48.4)	269 (50.1)	187 (45.9)	264 (48.4)	0.449

Patients with upper respiratory symptoms	644(43.3)	208(38.7)	195 (47.9)	241(44.2)	**0.016**

Patients with pneumonia	230 (15.4)	108 (20.1)	53 (13)	69 (12.7)	**0.001**

Fever	173 (11.6)	56 (10.4)	49 (12)	68 (12.1)	0.551

Cough	253 (17)	99 (18.4)	76 (18.7)	78 (14.3)	0.111

Sore throat	176 (11.2)	62 (11.5)	48 (11.8)	57 (10.5)	0.767

Shortness of breath	89 (6)	24 (4.5)	25 (6.1)	40 (7.3)	0.136

Myalgia	271 (18.2)	106 (19.7)	75 (18.4)	90 (16.5)	0.384

Joint pain	14 (0.9)	0	0	14 (2.6)	-

Loss of smell and taste	157 (10.5)	49(9.1)	58 (14.3)	50 (9.2)	**0.017**

Diarrhea	66 (4.4)	23 (4.3)	18 (4.4)	25 (4.6)	0.987

**Laboratory findings at the time of diagnosis** (Mean ± SS / Median min–max)					

White blood cell (cells/mm3)	5.8 ± 1.4	5.6 ± 1.4	5.8 ± 1.4	5.8 ± 1.5	0.078
	5.6 (1.6–15.2)	5.6 (1.6–15.2)	5.7 (3.1–12.6)	5.6 (2.3–13.3)	

Lymphocyte (cells/mm3)	2.2 ± 4	1.9 ± 2.5	2.2 ± 4	2.5 ± 5	0.075
	1.6 (0.3–47.8)	1.6 (0.3–30.3)	1.5 (0.5–41.7)	1.7 (0.5–47.8)	

Neutrophil (cells/mm3)	4.2 ± 7	4.2 ± 7.9	4.5 ± 7.3	4 ± 5.7	0.587
	3.3 (0.4–85.7)	3.2 (0.6–85.7)	3.4 (0.8–65.1)	3.3 (0.4–72)	

Glucose (mg/dL)	102 ± 32	104 ± 36	100 ± 28	100 ± 28	0.234
	96 (52–510)	98 (61–510)	96 (72–297)	96 (52–395)	

AST (U/L)	28.8 ± 21.8	29 ± 24.8	26 ± 15	30.7 ± 23	**0.024**
	23 (4–268)	23 (5–268)	22 (4–111)	25 (8–268)	

ALT (U/L)	28 ± 14.8	28 ± 16.2	26 ± 10.4	29.5 ± 16	**0.010**
	25 (9–214)	24 (9–161)	24 (11–74)	26 (12–214)	

LDH (U/L)	206 ± 68.3	211 ± 69	203 ± 65.1	202 ± 69	0.535
	199 (18–447)	202 (20–429)	195 (25–447)	200 (18–374)	

BUN (mg/dL)	26 ± 9.5	26 ± 9.5	25 ± 8.8	26.8 ± 10	0.172
	25.5 (2–65)	26 (8–65)	25 (2–55)	26 (4–58)	

Serum creatinine (mg/dL)	0.9 ± 0.19	0.9 ± 0.19	0.9 ± 0.17	0.9 ± 0.19	0.570
	0.9 (0.1–2.19)	0.9 (0.1–2.19)	0.9 (0.1–1.7)	0.9 (0.1–1.7)	

D-dimer (ng/mL)	295 ± 488.6	310 ± 576	304 ± 444	271 ± 393	0.871
	190 (1–9060)	204 (1–9060)	191 (3–444)	164 (1–4130)	

Ferritin (ng/mL)	84 ± 120.5	85 ± 116	76 ± 91	88 ± 144	0.606
	39.4 (3–1235)	37 (3–890)	36.9 (3–502)	44.4 (5–1235)	

Procalcitonin (ng/mL)	0.06 ± 0.1	0.06 ± 0.08	0.04 ± 0.03	0.08 ± 0.2	0.241
	0.03 (0.01–1.1)	0.04 (0.01–0.5)	0.03 (0.01–0.1)	0.03 (0.01–1.1)	

CRP (mg/dL)	8.8 ± 15.8	9.2 ± 17	9.7 ± 18.5	7.6 ± 11.8	0.085
	3.7 (0.5–156)	4.1 (0.5–156)	3.7 (0.1–135)	3.3 (0.1–90)	

**3th day of follow-up**					

At least one symptom	605 (40.6)	221 (41.2)	169 (41.5)	215 (39.4)	0.778

Fever	78 (5.2)	33 (6.1)	18 (4.4)	27 (5)	0.470

Cough	220 (14.8)	90 (16.8)	67 (16.5)	63 (11.6)	**0.029**

Sore throat	103 (6.9)	45 (8.4)	23 (5.7)	35 (6.4)	0.225

Shortness of breath	74 (5)	21 (3.9)	22 (5.4)	31 (5.7)	0.369

Myalgia	179 (12)	70 (13)	51 (12.5)	58 (10.6)	0.444

Joint pain	10 (0.7)	1 (0.2)	0	9 (1.7)	

Loss of smell and taste	169 (11.3)	57 (10.6)	44 (10.8)	68 (12.5)	0.582

Diarrhea	43 (2.9)	14 (2.6)	9 (2.2)	20 (3.7)	0.385

PCR positivity	(1213)	(432)	(322)	(459)	
	454 (37.4)	169 (39.1)	139 (43.2)	146(31.8)	**0.004**

**5th day of follow-up**					

At least one symptom	442 (29.7)	160 (29.8)	124 (30.5)	158 (29)	0.884

Fever	49 (3.2)	21 (3.9)	6 (1.5)	22 (4)	0.056

Cough	183 (12.3)	72 (13.4)	55 (13.5)	56 (10.3)	0.204

Sore throat	74 (5)	30 (5.6)	12 (2.9)	32 (5.9)	0.088

Shortness of breath	58 (3.9)	20 (3.7)	21(5.2)	17 (3.1)	0.267

Myalgia	137 (9.2)	54 (10.1)	41(10.1)	42 (7.7)	0.321

Joint pain	0	0	0	0	

Loss of smell and taste	116 (7.8)	40 (7.4)	33 (8.1)	43 (7.9)	0.925

Diarrhea	26 (1.7)	11 (2)	4 (1)	11 (2)	0.404

**14th day of follow-up**					

At least one symptom	239 (16.1)	105 (19.6)	68 (16.7)	66(12.1)	**0.003**

Fever	25 (1.7)	10 (1.9)	7 (1.7)	8 (1.5)	0.876

Cough	91 (6.1)	44 (8.2)	23 (5.7)	24 (4.4)	**0.031**

Sore throat	33 (2.2)	18 (3.4)	6 (1.5)	9 (1.7)	0.083

Shortness of breath	30 (2)	13 (2.4)	10 (2.5)	7 (1.3)	0.333

Myalgia	63 (4.2)	31 (5.8)	15 (3.7)	17 (3.1)	0.076

Joint pain	34 (2.3)	23 (4.3)	6 (1.5)	5 (0.9)	**0.001**

Loss of smell and taste	59 (4)	24 (4.5)	17(4.2)	18 (3.3)	0.599

Diarrhea	8 (0.5)	4 (0.7)	2 (0.5)	2 (0.4)	0.684

PCR positivity	(1105)	(389)	(298)	(418)	0.502
	35 (3.2)	9 (2.3)	11 (3.7)	15 (3.6)	

**30th day of follow-up**					

At least one symptom	180 (12.1)	70 (13)	61 (15)	49 (9)	**0.014**

Fever	26 (1.7)	12 (2.2)	10 (2.5)	4 (0.7)	0.059

Cough	56 (3.8)	21 (3.9)	21 (5.2)	14 (2.6)	0.115

Sore throat	22 (1.5)	8 (1.5)	7 (1.7)	7 (1.3)	0.830

Shortness of breath	26 (1.7)	9 (1.7)	11 (2.7)	6 (1.1)	0.180

Myalgia	57 (3.8)	29 (5.4)	14 (3.4)	14 (2.6)	**0.046**

Joint pain	37 (2.5)	12 (2.2)	11 (2.7)	14 (2.6)	0.913

Loss of smell and taste	38 (2.6)	15 (2.8)	15 (3.7)	8 (1.5)	0.088

Diarrhea	4 (0.3)	1 (0.2)	2 (0.5)	1 (0.2)	0.685

* BUN: Blood urea nitrogen, AST: Aspartateaminotransferase, ALT: Alanine aminotransferase, LDH: Lactate dehydrogenase, CRP: C-reactive protein, PCR: Polymerase chain reaction.

**Table 2:** Factors Affecting Symptom improvement in symptomatic outpatients on the 14th day of follow-up (n = 877)

Risk factors	OR_adj_ (95% confidence interval)	p value
**Absence of comorbidity**	1.9 (1.0–3.5)	0.054
**Absence of pneumonia**	1.4 (0.9–2.1)	0.145
**Antiviral treatment**		
Hydroxychloroquine	1.8 (1.2–2.7)	**0.005**
Hydroxychloroquine + Favipiravir	1.1 (0.7–1.7)	0.622
Favipiravir (Ref)	1	

**Table 3:** Factors Affecting PCR negativity on the 3th day of follow-up (n = 1489).

Risk factors	OR_adj_ (95% confidence interval)	p value
**Lack of symptoms**	1.41 (1.12–1.79)	**0.004**
**Absence of pneumonia**	1.57 (1.11–2.24)	**0.012**
**Antiviral treatment**		
Hydroxychloroquine	1.44 (1.09–1.91)	**0.010**
Hydroxychloroquine + Favipiravir	0.89 (0.66–1.19)	1.424
Favipiravir (Ref)	1	

**Table 4:** Factors affecting hospitalization of COVID-19 outpatients (n = 1489).

Risk factors	OR_adj_ (95% confidence interval)	P value
**Age (years)**	1.04 (1.01–1.07)	**0.018**
**Presence of comorbidity**	1.4 (0.6–3.2)	0.436
**Presence of any symptoms**	4.6 (2.0–10.7)	**<0.001**
**Antiviral treatment**		
Favipiravir	0.8 (0.3–2.2)	0.669
Hydroxychloroquine + Favipiravir	0.7 (0.3–2.2)	0.565
Hydroxychloroquine	Ref	
**Antiviral treatment in outpatients with pneumonia**		
Favipiravir	6.6 (2.4–18.4)	**<0.001**
Hydroxychloroquine + Favipiravir	7.3 (2.0–26.9)	**0.003**
Hydroxychloroquine	Ref	

## Footnotes

1 World Health Organisation (2020). Novel coronavirus-2019. Website (https://www.who.int/emergencies/diseases/novel-coronavirus-2019 [Accessed 15.12.2020]

2 T.C. Sağlık Bakanlığı COVID-19 Bilgilendirme Platformu (2020). Website https://covid19.saglik.gov.tr [Accessed 15.12.2020]
